# Parenting style in the NICU is stable, measurable, and predictive of 2-year parenting style

**DOI:** 10.21203/rs.3.rs-4693703/v1

**Published:** 2024-08-10

**Authors:** Mary Lauren Neel, Caitlin Kjeldsen, Rachelle Srinivas, Kevin McGovern, Zhulin He, Nathalie Maitre

**Affiliations:** Emory University School of Medicine; Emory University; Emory University School of Medicine and Children’s Healthcare of Atlanta

## Abstract

**Objective:**

To establish psychometric properties of Baby Care Questionnaire (BCQ) in preterm infants, individual level stability of BCQ scores from NICU to 2 years corrected gestational age (CGA), and to determine whether early BCQ scores predict 2-year parenting styles.

**Study design:**

In this prospective study, the BCQ assessed early parental structure and attunement at 4 time points between NICU and 2-years CGA. The Parenting Styles and Dimensions Questionnaire (PSDQ) at 2-years evaluated authoritative, authoritarian, and permissive parenting styles. Intraclass correlations analyzed reliability and Reliable Change Index (RCI) studied individual-level stability. Multivariate regression examined predictive properties.

**Results:**

n=162 parent/preterm infant dyads were followed sequentially. Cronbach’s α BCQ structure and attunement were 0.9 and 0.8. RCI showed high individual level stability of both constructs. Higher early structure scores were associated higher 2-year authoritative scores.

**Conclusions:**

Early parenting characteristics beginning in the NICU are stable in infancy, measurable, reliable, and predictive of 2-year parenting styles. Early parental structure correlates with 2-year authoritative parenting style.

## Introduction

One in ten children is born preterm(1) and is at increased risk for neurodevelopmental impairments and delays than their term counterparts.(2, 3) Parenting style is a modifiable environmental factor(4–6) associated with child outcomes.(7–10) Earlier parenting interventions have greater impact on outcome trajectories;(7, 11, 12) however, there are few measures of early parenting style in preterm infants, challenging design of interventions beginning as early as the Neonatal Intensive Care Unit (NICU). Furthermore, while studies generally suggest that parenting style between a parent and child is stable over time in the absence of interventions,(13–16) it has not been definitively established that parenting style beginning in the NICU is stable between a given parent/child dyad as the child matures and is discharged home. This is an important consideration in intervention design as we seek to measure the impact of interventions over time.

One of the few validated measures of early parenting style characteristics is the Baby Care Questionnaire (BCQ).(17, 18) Caregivers answer questions about their beliefs surrounding parental practices of feeding, sleeping, and soothing. The BCQ provides the caregiver with a score for attunement (reliance on infant cues/sensitivity) and structure (reliance on routines/consistency). A higher score indicates greater caregiver reliance on the construct. The BCQ has been validated from pregnancy through two years of age.(17, 18) Parental attunement and structure are generally associated with improved neurodevelopment in high-risk infants,(7, 9, 13, 16, 19, 20) and our previous studies highlighted the particular importance of parental structure to support positive adaptation and resilience in former preterm children.(9) However, no studies have examined associations between attunement and structure as measured by the BCQ and neurodevelopmental outcomes of these infants.

Parenting styles in parents of children beyond the first year of life can be more accurately measured because of increased opportunities for quantifiable interactions and validated tools to evaluate parenting style beyond infancy. Once commonly used tool is the Parenting Styles and Dimensions Questionnaire (PSDQ).(21) Caregivers answer questions regarding parenting practices using Likert scale responses. The PSDQ provides the caregiver three scores: a score for each authoritative, authoritarian, and permissive parenting styles. A higher score indicates increased use of that style by the respondent. The PSDQ has been widely used with caregivers of children from toddler-age through adolescence.(22) Many studies have shown associations between higher authoritative parenting style and improved behavioral and cognitive outcomes in term and preterm children.(16, 23–25) Authoritative parenting is considered the most balanced parenting style, whereby parents are both responsive and sensitive but also demanding, demonstrating developmentally appropriate expectations, structure, and consistency.(16, 23, 25) Conversely, parents who are demanding but not responsive are considered authoritarian parents and those who are responsive but not demanding are considered permissive. (16, 23, 25) Neither authoritarian nor permissive parenting styles are generally associated with optimal child developmental outcomes.(16, 26–28)

Thus, gaps exist regarding our ability to quantify early parenting style in preterm infants, the stability of early parenting style, and the association between early parenting style and well-established subsequent parenting styles. Therefore, we designed this prospective cohort study to examine: 1. the stability of individual level BCQ structure and attunement scores across multiple time points over the first 2 years of life, and 2. the ability of early structure and attunement to predict 2-year authoritative, authoritarian, and permissive parenting styles in parents of preterm infants. Given the current literature as described above, we hypothesized that structure and attunement would be stable for a given parent-child dyad over time. We further hypothesized that early structure would be associated with authoritative parenting, and early attunement would be associated with permissive parenting.

## Material/Subjects and Methods

### Design and participants

This prospective cohort study included infants born preterm (< 37 weeks gestation). Participants were recruited from a 130-bed level IV NICU in the midwestern United States. Infants were excluded if they had major congenital anomalies or remained endotracheally ventilated or on sedative medications at initiation of study procedures. Initial study procedures occurred between 34–36 weeks corrected gestational age (CGA). All study procedures and consents were approved by the hospital Institutional Review Board.

### Assessments and variables

Our study included assessments at four time points: 34–36 weeks, 3–4 months, 1 year and 2 years (all CGA). The first visit occurred in the NICU and the subsequent three visits occurred in the clinical research laboratory space or the neonatal follow-up clinic, depending upon family preference and availability. The self-identified primary caregiver completed the BCQ(17) at all four study timepoints. At the two year visit, the primary caregiver also completed the PSDQ.(21)

### Analysis plan

We used intraclass correlations (ICC) to analyze the reliability of structure and attunement, and Reliable Change Index (RCI)(29) to determine stability of individual level structure and attunement scores across multiple time points. We used a generalized linear mixed model to analyze if structure and attunement were distinct constructs. Finally, we use multivariate multiple regression with and without adjustment for gestational age at birth to examine associations between predictors (NICU and 3–4 month BCQ structure and attunement scores) and outcome (2-year PSDQ authoritative, authoritarian, and permissive scores). All BCQ and PSDQ scores were analyzed as continuous variables. Any instruments or surveys responses with >20% missing were omitted from analysis of the missing component. Analyses were conducted in R (R Version 2021).

## Results

Our study included n = 162 caregiver/infant dyads. Ninety-six percent of our caregivers self-identified as mother and four percent self-identified as father. Average infant gestational age at birth was 28 weeks, and caregiver education ranged from 7–11th grade to graduate education (Table 1).

Both structure and attunement showed high internal consistency on ICC with Cronbach’s alphas of 0.9 (CI) and 0.8 (CI), respectively. RCI also showed high individual level stability of both structure and attunement over time (Fig. 1). Over time, the percentage of dyads with no reliable change increases, such that between the NICU and 3–4 months, we see more dyads with reliable change than at later time intervals (Table 2). Generalized linear mixed models indicate that structure and attunement are independent constructs (see Supplemental Table 1).

Parental structure at 3–4 months was associated with authoritative parenting style at 2 years (p < 0.05). Parental attunement in the NICU and at 3–4 months was associated with permissive parenting style at 2 years (p < 0.1). Parental attunement in the NICU was associated with authoritarian and authoritative parenting style at 2 years (p < 0.05, p < 0.1, respectively) (Table 3). Significant associations between predictors and outcomes did not change with or without adjustment for gestational age (Supplemental Table 2).

## Discussion

Our results show that individual parental structure and attunement are measurable and generally stable over the first two years of life in this cohort of preterm infants and their parents. A limiting factor to date in parenting intervention research for high-risk neonates has been lack of effective measures of early parenting style.(17) Our results support the reliability of the BCQ(17, 30) for use in the parents of preterm infants, even as early as the NICU. Quantifiable measures of early parental structure and attunement that are stable over time between a given parent and child are critical to identify dyads at risk for suboptimal interactions and to measure the effectiveness of parenting interventions.

Furthermore, these results elucidate two important and nuanced aspects of the parenting literature. First, the literature broadly suggests that parenting style between a parent and child is stable over time in the absence of high-quality interventions.(13–16) Our results support this assertion from the NICU onwards, and specifically add that parental structure and attunement between parent and preterm child are stable over the first two years of life. Second, the parent and child constitute a dyad with bidirectional interactions, although the preponderance of evidence is that in the early years, the parent influence on the child predominates.(7, 8, 19) Our results add to the evidence suggesting the parent as the major early contributor to the dyad even for preterm infants with prolonged hospital stays: parenting style remained stable over the first two years of life even as the child matured in the NICU, was discharged from the hospital, and achieved new developmental milestones and competencies in the home.

Though our results suggest that parental structure and attunement are overall stable over the first two years of life in this cohort, a greater number of dyads showed reliable change between the NICU and 3–4 months than at subsequent time intervals (Table 2). Perhaps parenting within the dyad has not yet been cemented at this earliest time interval, offering a window of opportunity between NICU discharge and the first months at home during which parenting interventions may be most effective. This assertion is consistent with previous work supporting greater effectiveness of early interventions to optimize parent-child interactions.(7, 14, 31, 32)

Higher early parental structure was associated with higher 2-year authoritative parenting scores. Much of the research on parenting style in preterm infants has focused on parental responsivity (sensitivity);(7) however, recent studies have suggested the importance of parental structure for promoting resilience and improved outcomes in preterm infants,(9, 16) Parental structure includes predictability, consistency, follow-through, and scaffolding for learning.(7, 9, 13, 16, 33) This study adds that structure is a component of early parenting that correlates with a future supportive parenting style (authoritative) for preterm infants. Excitingly, parental structure can be measured in parents of infants before it is feasible to measure authoritative parenting style using tools designed for older children.(21)

Associations between early parental attunement and 2-year parenting style are more complicated. Parental attunement is similar to parental responsivity/sensitivity, a construct extensively studied in the preterm parenting literature.(7) Parental responsivity is associated with improved cognitive and behavioral outcomes in formerly preterm infants;(13, 19, 34) thus, it is unsurprising that our results also show associations between higher early parental attunement and higher 2-year authoritative parenting scores. However, parental responsivity is only part of an optimal parental approach and is best leveraged when balanced with demandingness and structure.(7, 13, 16, 23, 25) The association between higher early parental attunement and higher 2-year permissive parenting scores may be explained by this imbalance. An overreliance on early parental attunement may translate to a continued overreliance on parental responsivity, unbalanced by parental structure and demandingness, potentially promoting permissive parenting style at 2 years. Parental difficulty providing developmentally appropriate expectations, structure, and consistency for formerly preterm children is commensurate with literature on “vulnerable child syndrome (VCS).”(16) In VCS, parents perceive their child as more medically, socially, or developmentally fragile than is objectively justified by the child’s current state.(35) VCS is more common in parents of children who have experienced a significant medical illness or event, such as prematurity. VCS can precipitate maladaptive parenting styles, such as permissive parenting, that are associated with worse child cognitive and behavioral outcomes.(16, 35)

Less intuitively, our study suggests that early parental attunement predicts higher 2-year authoritarian parenting scores. To further explore this seemingly contradictory result, two reviewers (MLN, CPK) performed an independent question-by-question comparison between the questions and constructs of the BCQ and the PSDQ followed by discussion of rare discordance (supplemental Fig. 1). Concordance was high between the two reviewers with discordance on only 5% of questions that were discussed until consensus was reached. Early parental attunement and structure assessed by the BCQ did not map well onto authoritarian parenting style assessed by the PSDQ. As described, the BCQ specifically examines parenting constructs in parents of infants. While structure and attunement are components of authoritative, authoritarian, and permissive parenting styles in parents of older children, authoritarian subfactors of physical coercion and verbal hostility in particular(21) are not represented on the BCQ given the young age of the infants. Thus, we likely cannot draw valid conclusions about 2-year authoritarian style using BCQ predictors given lack of overlap on question-by-question construct mapping (supplemental Fig. 1). However, BCQ attunement and structure do map well to PSDQ authoritative and permissive parenting style constructs (supplemental Fig. 1).

Limitations: As discussed above, these results do not allow us to draw conclusions about early predictors of authoritarian parenting style. Secondly, as with all parenting research in infants, we were limited by the early parenting constructs we could measure with established tools. While structure and attunement are key components of early parenting, parenting style has a multiplicity of dimensions.(13) Thus, more validated tools are needed to assess the full dimensionality of early parenting style. Thirdly, for practical considerations, we evaluated parenting style at 2 years, but future work could extend our conclusions to preschool age when an important transition in the participatory environment occurs. Finally, this study focused on parental contribution to the dyad but future work could examine child contributions and/or dyadic interactions as well.

## Conclusions

Early parental attunement and structure are stable, measurable, reliable, and predictive of 2-year parenting style in parents of preterm infants. Early parenting style is stable in the absence of intervention.(14, 15) Fortunately, parenting can be modified with high quality interventions and the effectiveness of parenting interventions are not modified by sample demographics.(36) The earlier the intervention, the greater the impact on outcomes.(11, 12) To date, most work in parents of preterm infants has focused on attunement,(7) but this is likely only one aspect of the optimal approach. Parental structure is a component of early parenting that correlates with a future supportive parenting style for preterm infants. These results suggest opportunities to design early parenting interventions targeting parental structure to support the best developmental and behavioral outcomes for our preterm infants.

## Figures and Tables

**Figure 1:** Reliable change indices show high individual level stability of structure and attunement over time Legend: Intraclass correlations for structure and attunement were 0.9 and 0.8, respectively. These intraclass correlations were used to calculate Reliable Change Index for individual-level stability. The top three figures show the Reliable Change Index for structure, and the bottom three figures show the Reliable Change Index for attunement. Both structure and attunement show high individual level stability over time, although more dyads showed reliable change between the NICU and 3–4 months than at subsequent time intervals.

## Data Availability

Non-identifiable data will be available promptly on reasonable request from the corresponding author up to 5 years after study conclusion.
